# Evolutionary origin of lubricated joints at the dawn of jawed vertebrates

**DOI:** 10.1371/journal.pbio.3003044

**Published:** 2025-02-26

**Authors:** J. Gage Crump

**Affiliations:** Eli and Edythe Broad CIRM Center for Regenerative Medicine and Stem Cell Research, Keck School of Medicine of the University of Southern California, Los Angeles, California, United States of America

## Abstract

The evolutionary origin of the lubricated joints that allow our interconnected skeleton to swivel, rotate, and bend has remained a mystery. This Primer explores a new comparative study of joints in PLOS Biology which points to lubricated joints arising in the earliest jawed vertebrates.

## Teaser

The evolutionary origin of the lubricated joints that allow our interconnected skeleton to swivel, rotate, and bend has remained a mystery. A new comparative study of joints in *PLOS Biology* points to lubricated joints arising in the earliest jawed vertebrates.

Our skeletons are made up of many interconnected bones, such as in our limbs and faces. The ability of these bones to freely move in relation to one another allows an extraordinary range of motion in activities such as running, climbing, and eating. This flexibility is facilitated by specialized synovial joints between bones, which are lined by squishy cartilage and filled with lubricating fluid. While it had been shown that synovial joints likely exist in all bony vertebrates [1], when these types of joints first arose during evolution was less clear. By studying joints in cartilaginous fishes and the jawless vertebrates lamprey and hagfish, work from the lab of Neil Shubin in *PLOS Biology* [2] provides evidence that synovial joints first arose around the same time that the earliest vertebrates acquired hinged jaws.

For a long time, the textbook view was that complex synovial joints first evolved in early tetrapod limbs to accommodate the increased gravitational loads associated with walking on land. However, fishes clearly also bear high loads in their fins and jaws during predation and swimming. As early as the 1940s, anatomists such as Haines had recognized that the jaw joints of the bony fishes gar and sturgeon, as well as the rat fish (belonging to the chimeras, one of the 3 major groups of cartilaginous fishes), had a diarthrodial (i.e., synovial-like) morphology [3]. Davies had made similar observations in a different group of cartilaginous fishes, the skates [4]. More recently, molecular and genetic evidence confirmed the synovial nature of both jaw and fin joints in zebrafish [1]. In Sharma and colleagues, the authors now show that cartilaginous fishes (the little skate and bamboo shark) have bona fide synovial joints. Histology and micro-computed tomography reveal a clear synovial cavity within the jaw and pelvic joints, and cartilage cells lining these joints have a distinct proteoglycan and collagen composition from the deeper cartilage layer. In addition, as with tetrapod joints, little skate joints are enriched for Wnt [5] and Gdf5 [6] signaling and require muscle contraction for cavity formation, although Wnt [7] and Gdf5/6 [8] pathways have also been implicated in the development of non-synovial cartilaginous joints, such as those of the intervertebral discs. Cartilaginous fish are thought to have descended from bony fish that secondarily lost bone and their cartilage has a rigid calcified matrix. Given the terrifying bite forces of sharks, it would therefore make sense that synovial joints would also be important in providing flexibility to their rigid cartilaginous skeletons.

If synovial joints are in all living jawed vertebrates examined, did they first evolve in the jaw? Reanalysis of well-preserved fossils of early vertebrates does suggest that emergence of synovial joints coincides with that of jaws. Jawless osteostracans lived about 400 million years ago during the Silurian and Devonian periods and had pectoral fins that likely allowed them to be good swimmers. However, the bones connecting their pectoral fins and head shields were filled with many canals that may have allowed nerves and blood vessels to penetrate the joint cavity, suggesting it was not fluid-filled. In addition, bones with canals extending to the joint surface would not have been conducive to containing fluid in a joint cavity. In contrast, fossils of an early jawed vertebrate, the placoderm, show an articulated joint between the pectoral fin and girdle with the canals not penetrating to the joint surface, consistent with the potential presence of a fluid-filled joint cavity. Yet, as cartilage rarely survives the fossil record, direct evidence for cartilage-lined synovial joints in placoderms is lacking. To further address the types of joints in jawless vertebrates, the authors also examined the cartilaginous skeletons of the only 2 extant agnathans, lamprey and hagfish. Micro-computed tomography and histology revealed that their joints were filled with tissue rather than having a fluid-filled cavity, and although proteoglycans were present in their cartilages they were not concentrated at joint surfaces. These findings suggest that modern agnathans lack synovial joints, which the authors suggest may not be needed given the soft, flexible nature of their cartilages.

**Fig 1:**
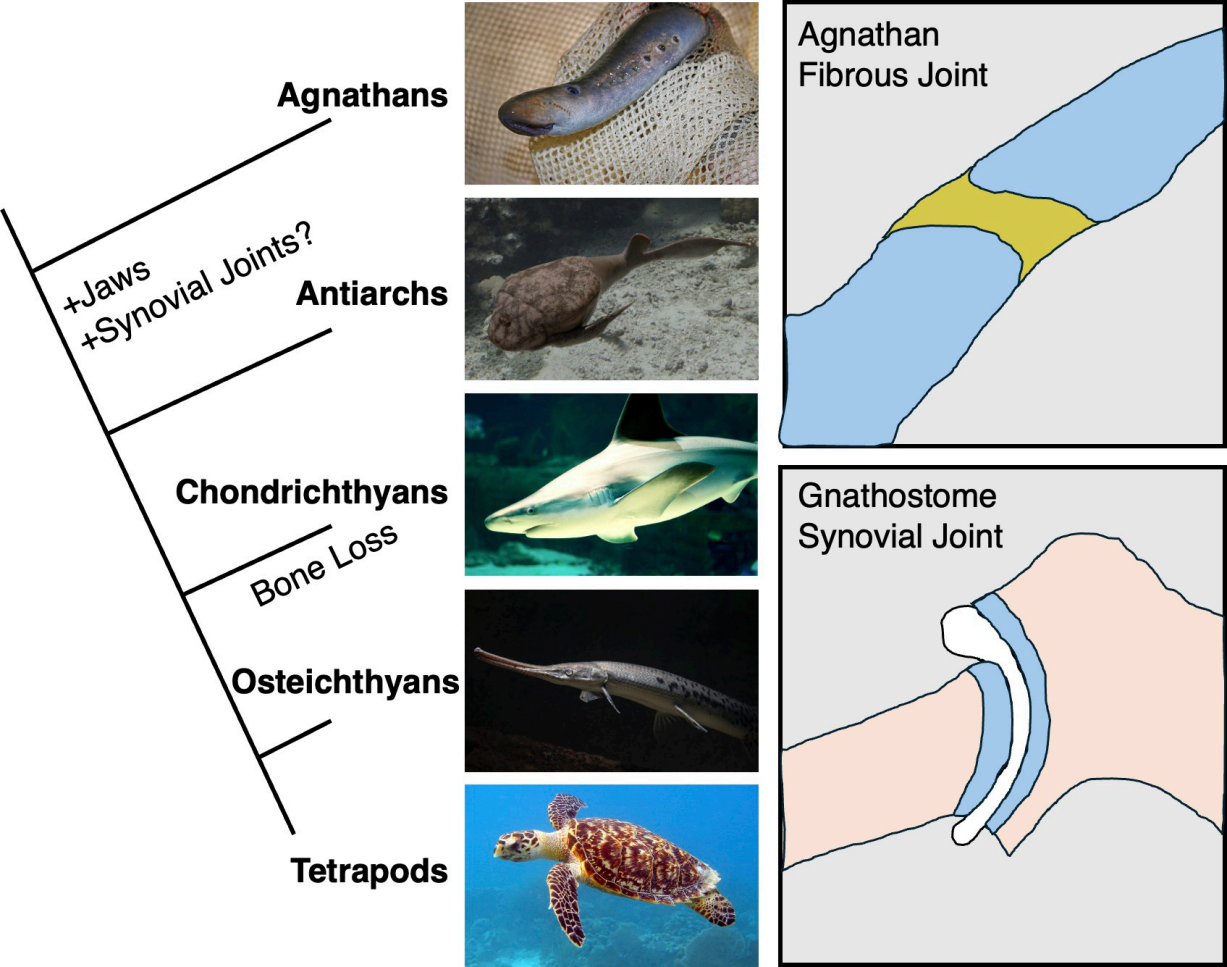
Emergence of synovial joints in early jawed vertebrates. Agnathans such as lampreys lack synovial joints. The first putative evidence of synovial joints in the fossil record is in early gnathostomes such as the antiarch placoderm fish that lived during the Silurian and Devonian periods. Modern gnathostomes such as cartilaginous fishes (i.e., chondrichthyans such as the brown shark), bony fishes (i.e., osteichthyans such as the longnose gar), and limbed vertebrates (i.e., tetrapods such as the Hawksbill turtle) possess synovial joints in their jaws, fins/limbs, and other locations. All images used are freely available without copyright restrictions. At top right, a typical agnathan joint is shown where glycosaminoglycans and proteoglycans are uniformly distributed across the cartilages (blue) that are connected by fibrous tissue (olive). At bottom right, a typical gnathostome synovial joint is shown where surface cartilage has a unique proteoglycan composition from the underlying cartilage and bone, and a fluid-filled cavity separates adjacent skeletal elements.

The finding that cartilaginous fishes also have synovial joints provides new opportunities to understand core mechanisms of joint formation, including cavitation. It will be important to understand the extent to which features of joints in bony vertebrates are conserved in cartilaginous fishes. In bony vertebrates, joints are stratified into distinct layers, culminating in bone at the base of successive cartilage layers. Given that cartilaginous fishes lack the types of growth plates seen in endochondral bones, it will be interesting to understand how the development and function of their joints are integrated with the underlying calcified cartilage. It will also be informative to investigate broader molecular similarities of joints across vertebrates, such as whether joints in cartilaginous fishes contain synovial fibroblasts and express the key lubricating protein Lubricin/Proteoglycan 4 [9]. From an evolutionary perspective, the findings reveal a correlation between the first appearance of jaws and synovial joints (**Fig 1**). However, this is largely based on circumstantial inferences from only a handful of fossils and analysis of the only 2 living types of agnathans, lamprey and hagfish, which are likely highly derived from their agnathan predecessors. More fossil evidence will therefore be needed to establish whether synovial joints first evolved to provide increased mobility to jaws, or whether a synovial-like character emerged alternatively or simultaneously in fins or other articulated skeletal structures. In addition, further molecular genetic comparisons between modern agnathans and gnathostomes may help reveal the key changes to gene structure and regulation that resulted in formation of a distinct type of cartilage at the joint surface and creation of a lubricated cavity. Lastly, understanding whether the joints of cartilaginous fishes regenerate, as has recently been shown in zebrafish [10], may lead to new strategies to reverse degenerative joint changes in arthritis.
